# *Die Kämpfe únd schláchten*—the struggles and battles of innate-like effector T lymphocytes with microbes

**DOI:** 10.3389/fimmu.2023.1117825

**Published:** 2023-04-24

**Authors:** Sebastian Joyce, Gosife Donald Okoye, John P. Driver

**Affiliations:** ^1^ Department of Veterans Affairs, Tennessee Valley Healthcare Service, Nashville, TN, United States; ^2^ Department of Pathology, Microbiology and Immunology, The Vanderbilt Institute for Infection, Immunology and Inflammation and Vanderbilt Center for Immunology, Vanderbilt University Medical Center, Nashville, TN, United States; ^3^ Division of Animal Sciences, University of Missouri, Columbia, MO, United States

**Keywords:** NKT (natural killer T) cell, MAIT (mucosal-associated invariant T) cell, innate-like effector lymphocyte, symbionts, pathobiont

## Abstract

The large majority of lymphocytes belong to the adaptive immune system, which are made up of B2 B cells and the αβ T cells; these are the effectors in an adaptive immune response. A multitudinous group of lymphoid lineage cells does not fit the conventional lymphocyte paradigm; it is the unconventional lymphocytes. Unconventional lymphocytes—here called innate/innate-like lymphocytes, include those that express rearranged antigen receptor genes and those that do not. Even though the innate/innate-like lymphocytes express rearranged, adaptive antigen-specific receptors, they behave like innate immune cells, which allows them to integrate sensory signals from the innate immune system and relay that umwelt to downstream innate and adaptive effector responses. Here, we review natural killer T cells and mucosal-associated invariant T cells—two prototypic innate-like T lymphocytes, which sense their local environment and relay that umwelt to downstream innate and adaptive effector cells to actuate an appropriate host response that confers immunity to infectious agents.

## Introduction: ‘For a secret offence, a secret revenge’

This subtitle ‘For a secret offence, a secret revenge’ (see **Box 1**) exemplifies the metaphorical descriptions of *fin-de-siècle*—turn of the 19th century, scientific discoveries written for the benefit of the general public; this style, quite common then and in the early 20th (3, 5), remains in textbooks and lectures in pathology, microbiology, and immunology. By that time, many—Antony van Leeuwenhoek (6), Robert Hooke (7), Theodor Schwann (8), and Matthias Schleiden (9), had independently peered down the microscope, developing the ‘cell theory’—the cell as the fundamental unit of life. Now entered Rudolf Virchow (10) who espoused ‘*omnis cellula e cellula*’—every living cell derives from another cell, the melodic phrase coined by François-Vincent Raspail (11)—from observations of *leukocythemia*—leukemic cells in the blood of a 50-year-old woman and formed the cellular basis of disease (10, 12). Robert Koch and Louis Jean Pasteur independently developed the microbial basis of infectious disease (13), and Élie Metchnikoff (previously Ilya Ilyich Mechnikov) whose astute observations of cells swarming toward the splinter prick in the starfish larva and their attempts to eat it, voraciously gnawing at it—that is termed phagocytosis, birthed cellular immunology (5, 14, 15), while from the opposing and warring Paul Ehrlich school originated humoral immunity (15–17).

**BOX 1 *Fin-de-siècle*—a turn-of-the-19th-century metaphorical description of the defense system as a warring system of the body that restores balance when tipped over by an infection.**

The subtitle ‘For a secret offence, a secret revenge’ owes to the title of one of the fables in ‘*Vacation Stories: Five Science Fiction Tales*’ written by the 1906 Nobel Laurate Santiago Ramón y Cajal, published originally in the Spanish language assuming the alias of ‘Dr. Bacteria’. These fables were written for Cajal’s scientific friends. Famed for the ‘neuron doctrine’ and precise and beautiful drawings of the nervous system (1), Cajal is less known for his artistic and literary works because much of these cultural contributions were poorly recorded and archived. Cajal “*wrote a collection of twelve fables or semi-philosophical, pseudoscientific tales that* [*I*]” he “*never dared take to press, both for the oddness of their ideas and the laxity and carelessness of their style* (2).” Fortunately, the collection of five science fiction works have survived Cajal and time in ‘Vacation Stories’; the remaining seven “*sleep the slumber … far deeper than the so-called* sleep of slumber[.]” not as “*failed artistic works*” as Cajal’s Preface would make the reader to believe (2) but rather because those manuscripts were never found (3, 4).

Viewed against this historic backdrop, ‘for a secret offence, a secret revenge’ refers to the body’s elegant defense system working against agents that cause infectious diseases—the battles raged between immune cells and bacteria. The immune system is generally described as a warring system that oftentimes wins battles yet may lose a war: the morbidity and mortality caused by severe acute respiratory syndrome coronavirus 2 infection is a sorry reminder of the perils of the warring immune system. While it is a warring system indeed, it does not attack indiscriminately. The immune system has learnt over eons to coexist with billions and zillions of bacteria and other microbes in a symbiotic habit.

Amid *kämpfe únd schláchten* with microbes and other forms of external (irritants and allergens) and internal (mutant cells and metabolic toxicants) dangers, in complex multicellular metazoans arose a sensing-and-actuating system—the immune system. In vertebrates, the initial response to aforementioned dangers is actuated by the older innate immune system. In vertebrates, the innate immune system, which arose in early metazoan faunas—the simple invertebrates, is made entirely of the myeloid lineage of hematopoietic cells such as macrophages, dendritic cells and mast cells in tissues and by monocytes, neutrophils, basophils, and eosinophils patrolling the blood and, on demand, tissues as well. As the innate immune system responds to danger, it alerts the adaptive immune system, which kicks into full gear should the innate immune response not restore the host’s altered *milieu intérieur* (homeostasis) to its original state—or close to it. The adaptive system is slow in acting and is made entirely of lymphoid lineage cells. These cells sense alterations in the homeostatic state with the use of antigen-specific receptors encoded by somatically rearranged gene segments, clonally expressed by B and T lymphocytes—the B-cell receptor (BCR) and αβ T-cell receptor (TCR). Such B and T lymphocytes together constitute the conventional lymphocytes. The clonal expression of BCR and TCR requires the priming of the adaptive immune system by either immunization with antigen or natural infection for the clonal expansion of the low-frequency antigen-specific lymphocytes to clear infections and to protect against infectious diseases. This requirement for priming distinguishes the adaptive immune system from the innate, which reacts quickly, without the need for prepriming.

**Figure 1:**
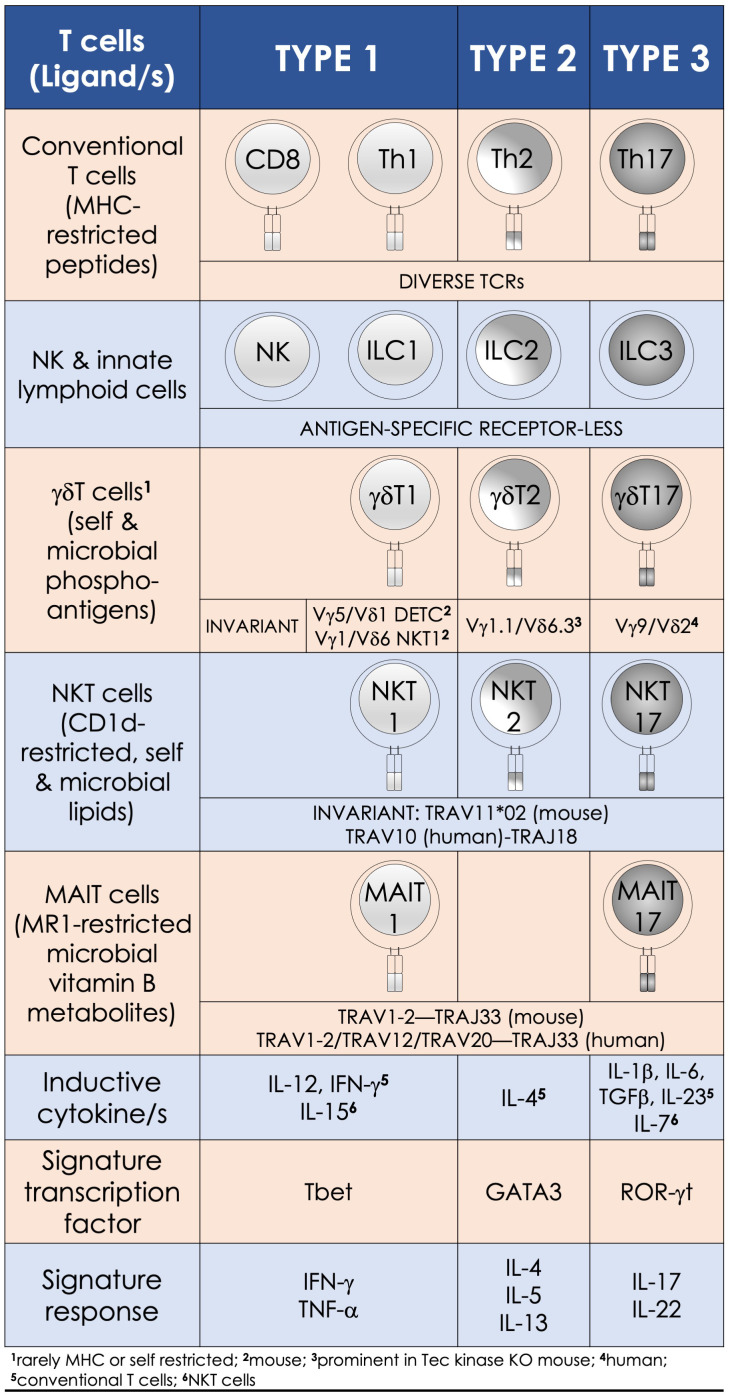
Innate-like effector lymphocyte functions mirror type 1, type 2, and type 3 effector cells. Natural killer T (NKT), mucosal-associated invariant T (MAIT), and γδT cells are characterized by semi-invariant T-cell receptor (TCR) expression by contrast to conventional T cells express a diverse TCR (IMGT nomenclature) repertoire. By contrast, innate lymphoid cells and NK cells do not express rearranged antigen receptors. Type 1 effectors include the cytotoxic NK and CD8^+^ T cells and T helper (Th) 1 cells, as well as NKT1, MAIT1, and γδT1 cells. They require IL-12 for induction, which is bolstered by IFN-γ. T-bet and the related eomesodermin transcription factors control the differentiation of type 1 effector cells, which are essential for immunity against intracellular pathogens. Type 2 effector cells include Th2, NKT2, and γδT2 cells. These cells are activated by IL-4 and require GATA3 for their effector differentiation. Their physiologic functions—e.g., parasite expulsion, and pathologic—e.g., airway hypersensitivity, are mediated by IL-4, IL-5, and IL-13 secretions. RORγt—the lineage specific transcription factor program type 3 effectors, which include Th17 and NKT17, MAIT17, and γδT17 cells. Lineage-specific inducive factors include IL-6, TGF-β, IL-1β, IL-23, and IL-7. Type 3 effector cells secrete IL-17 and IL-22 upon activation, by which they mediate tissue repair and confer immunity to extracellular bacteria and fungi.

Circa 1973, a non-B, non-T—the ‘null’ killer lymphocyte, which could kill tumor cells without prior priming of the immune system, was discovered. Now called natural killer (NK) cells, their discovery alerted to lymphocytes that behave like the cells of the innate immune system and featured the quiet annunciation of unconventional lymphocytes (18). Next, a decade later, the start of the year 1983 unveiled with the discovery of B lymphocyte subsets: one that secreted natural antibodies (B1a) and the other that produced antibodies to bacterial polysaccharides and T lymphocyte–independent antigens (B1b) in addition to the conventional B2 B cells of the adaptive immune system (19). Then in ca. 1986 came the discovery of γδ T cells, which express the γδ TCR genes—a kin to the αβ TCR (20). The ensuing decades announced the discovery of many more unconventional lymphocytes (**Figure 1**): e.g., natural killer T (NKT) cells, mucosal-associated invariant T (MAIT) cells, mouse CD8αα intraepithelial T lymphocytes, mouse H-2M3-restricted T cells, mouse/human H-2Qa1/HLA-E-restricted T cells, and human group 1 CD1-restricted T cells as well as lymphoid tissue inducer cells and innate lymphoid cells [reviewed in refs (21, 22).]. This collection of unconventional T lymphocytes we here call innate/innate-like effector lymphocytes.

**Figure 2:**
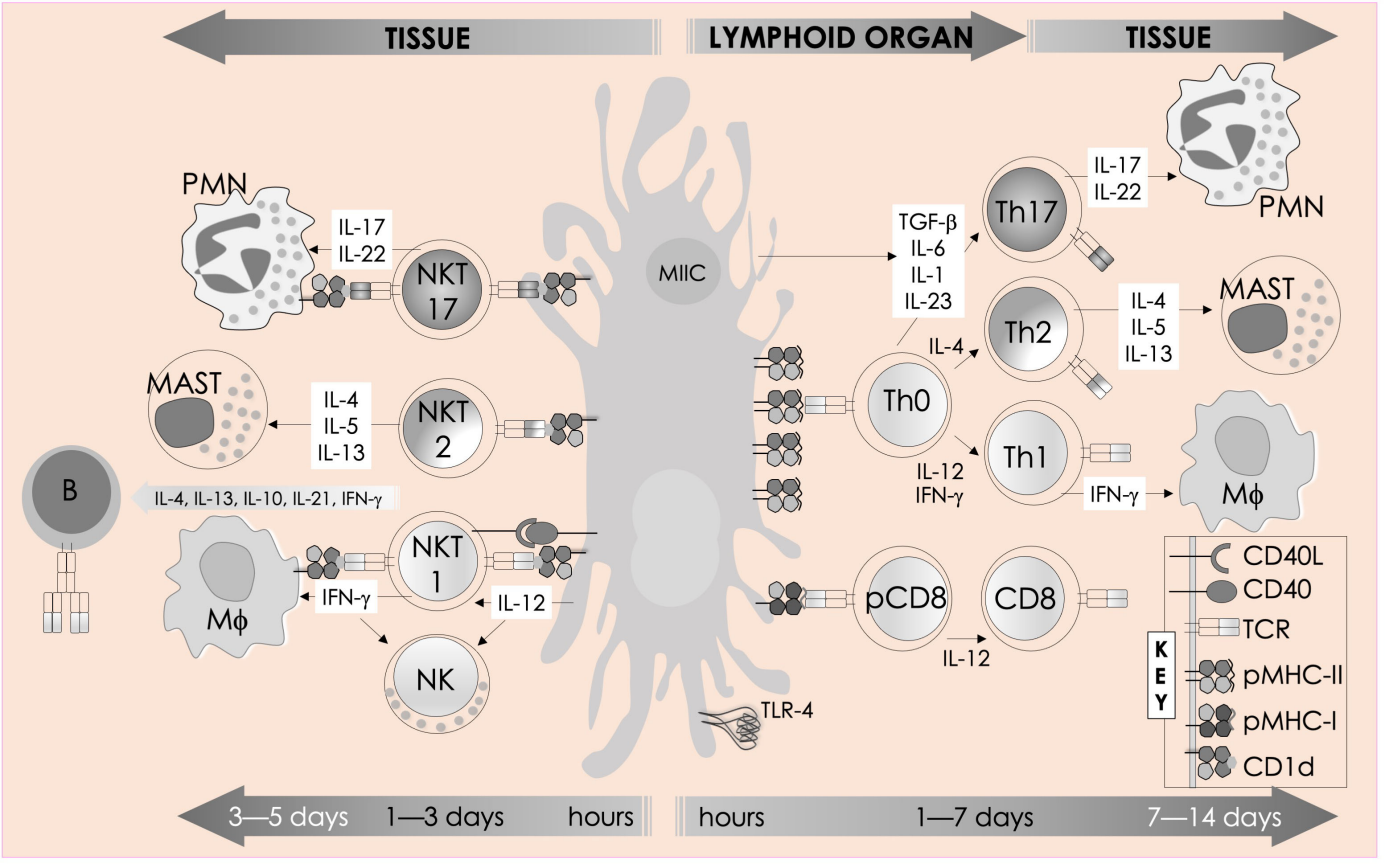
Immune functions of mouse NKT cells. NKT cell activation is initiated by semi-invariant NKT cell receptor interactions with cognate antigen and bolstered by costimulatory interactions between CD28 and CD40 and their cognate ligands CD80/86 (B7.1/7.2) and CD40L, respectively. The resulting activated NKT cells crosstalk with members of the innate and the adaptive immune systems by deploying cytokine and chemokine messengers. Upon activation *in vivo*, NKT cells rapidly secrete a variety of cytokines and chemokines, which influence the polarization of CD4^+^ T cells toward Th1 or Th2 cells as well as the differentiation of precursor CD8^+^ T cells to effector lymphocytes, and B cells to antibody-secreting plasma cells. Some of these mediators facilitate the recruitment, activation, and differentiation of macrophages and DCs, which results in the production of interleukin (IL)-12 and possibly other factors. IL-12, in turn, stimulates NK cells to secrete IFN-γ. Thus, activated NKT cells have the potential to enhance as well as temper the immune response. This schematic rendition is an adaptation of past reviews (35, 37–41) and works cited in the text.

The multitudinous innate/innate-like effector lymphocytes share several common features. In addition to being of lymphoid origin, they act quickly as they display a memory phenotype similar to antigen-experienced conventional lymphocytes yet, unalike conventional lymphocytes, retain no memories of past pathogen encounters. After development, innate/innate-like effector lymphocytes become home to secondary lymphoid and/or nonlymphoid tissues. They are stationed at barrier sites where the microbial consortia are known to congregate (19, 23, 24). As discussed below in the “Hygiene Hypothesis” section, products from these consortia facilitate the development and/or maturation of a subset of innate/innate-like effector lymphocytes (25–29). Those innate-like lymphocytes that express rearranged BCRs or αβ/γδ TCRs recognize their cognate ligands by germ-line encoded portions of the antigen-specific receptors using an innate recognition logic (30–34). Innate/innate-like lymphocytes react to self- and nonself-ligands: Some recognize H-2Qa1/HLA-E-restricted self and/or microbial peptides, H-2M3-restricted *N*-formylated mitochondrial/microbial peptides, group I and group II CD1-restricted lipids—e.g., αβ and γδ T cells and NKT cells, or major histocompatibility complex (MHC)–related 1 (MR1)-restricted metabolites—e.g., MAIT cells. Others recognize ligands directly without the need for MHC/non-MHC restricted presentation—e.g., intact proteins, small molecules/phosphometabolites—e.g., γδ T cells, or phospholipids—e.g., B1a cells and γδ T cells. Further, inflammatory cytokines alone—e.g., type I interferons (IFNs) or interleukin (IL)-12 and IL-18 by themselves—without the need for antigenic or agonistic ligands, can activate innate-like T lymphocytes. Innate/innate-like effector lymphocytes are quick responders; they can act as quickly as cells of innate immune system or faster [reviewed in refs (21, 22)]. This feature in several innate/innate-like effector lymphocytes is ingrained during development by a genome regulatory network under the control of a promyelocytic leukemia zinc finger transcription factor (encoded by *Zbtb16*; reviewed in ref (35, 36)]. Activated innate/innate-like effector lymphocytes secrete a wide variety of cytokines and chemokines with which they can steer downstream type I, II, and III immune responses (**Figure 2**). Thereby, they integrate sensory output/s received from the innate immune system to provide context to downstream innate and adaptive immune responses (35, 42). Here we review how NKT cells and MAIT cells—two prototypic innate-like T lymphocytes, sense their local environment and relay that umwelt to downstream innate and adaptive effector cells to actuate an appropriate response that confers protection from infectious diseases.

## Natural killer T and mucosal-associated invariant T cells—two peas in a pod

There are multiple types of NKT and MAIT cells that are distinguished by their αβ TCR usage and, consequently, the ligands they recognize (21). The focus in this review is on NKT and MAIT cells that express an invariant TCR α-chain: semi-invariant NKT cells begotten from the rearrangement of TRAV11*02 (mouse Vα14i) or TRAV10 (human Vα24i) to TRAJ18 and MAIT cells from TRAV1-2 (mouse and human Vα9i and human TRAV12/TRAV20) to TRAJ33 rearrangement [reviewed in refs (35, 43–45)]. A curious feature of these rearrangements is not only the conserved TRAV to TRAJ usage but also that this rearrangement results in conserved residues that make up the CDR3α (complementarity determining region 3α) loop of the TCR α-chain. Furthermore, invariant Vα14i α-chain pairs with TRBV13-2*01 (Vβ8.2), TRBV29*02 (Vβ7), or TRBV1 (Vβ2) β-chain to form a functional mouse semi-invariant NKT cell TCR. Additionally, the Vα24i α-chain pairs with the mouse TRBV13-2*01 orthologue—TRBV25-1 (Vβ11) to form a functional human semi–invariant NKT cell TCR. Akin to the semi-invariant NKT cells, MAIT cells pair with a limited set of β-chains to form a functional MAIT cell TCR. The conserved nature of the functional NKT and MAIT cell TCRs allow them to recognize their respective ligands—CD1d+lipid/s and MR1+vitamin metabolites, respectively, by means of conserved interactions—i.e., with an innate-like recognition logic (reviewed elsewhere: refs (29, 30, 34)].

In a similar vein, the pig semi-invariant NKT cells use the pTRAV10 TCR Vα gene segment, which is highly homologous to segments encoding human TRAV10, mouse TRAV11, and rat TRAV14S1—the canonical Vα segments used by the semi-invariant NKT cells in these species. The best alignments for pTRAJ18*01 were TRAJ18, TRAJ18, and TRAJ18, the Jα18 gene segments used by the human, rat, and mouse invariant α-chain, respectively. pTRBV25 is most similar to human TRBV25-1 (Vβ11), mouse TRBV13-2*01 (Vβ8.2), and rat Vβ8.2—the canonical Vβ segments used by the semi-invariant NKT cells in these species (46).

NKT cell functions are controlled by a variety of lipid agonists presented by CD1d molecules. These agonists include glycosphingolipids such as α-galactosylceramide (αGalCer) and α-glucosyldiacylglycerols and related compounds—both of host/self and microbial origins (see **Table 1** and references therein). MAIT cell functions are controlled by metabolites in the riboflavin biosynthesis pathway when presented by MR1 (43, 44, 59–62). One such MAIT cell agonist is a derivative of vitamin B2 metabolite 5-(2-oxopropylideneamino)-6-d-ribitylaminouracil (5-OP-RU), which is synthesized by both symbiotic and pathogenic bacteria (43, 44, 59, 60). Consequently, infections with bacteria- harboring mutations in the *rib* gene/s prevent MAIT cell activation, which in some infections can prove fatal (62).

**Table 1:** Natural, synthetic, microbial, and self natural killer T (NKT) cell agonists: structures and properties.

Lipid (class^1^) origin	Chain length^2^	Structure	Agonist activity ^3,4^	Ref.
Natural and synthetic				
Agel 9b (GSL) The sponge *Agelas mauritianus*	C17 (C_16_-Me) phyto C24	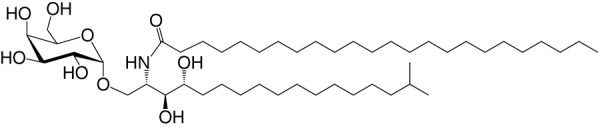	Antitumor	(47, 48)
KRN7000 αGalCer (GSL) synthetic analogue of Agel 9b	C18-phyto C26	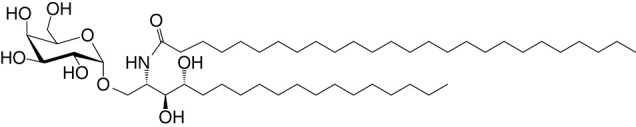	Very strong; robust IFN-γ, IL-4, and other cytokines	(49)
Microbial				
αGalUCer (GSL)	C18-phyto C14	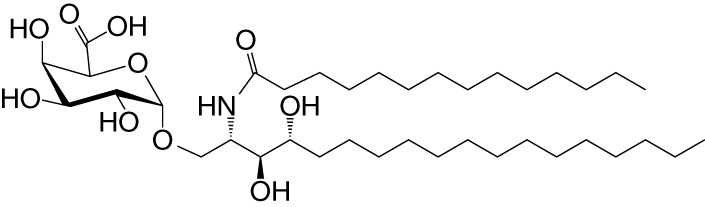	Weak; *Sphingomonas* spp.	(50, 51)
Asp B (GSL) *Aspergillus fumigatus*	C20:2-C_9_ Me C16-C_2_ OH	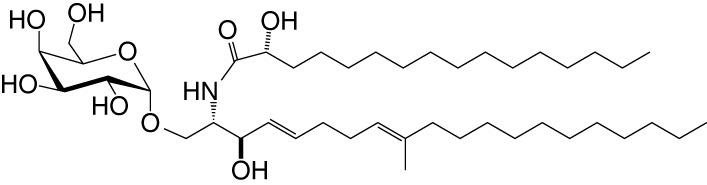	Weak	(52)
Acyl-αGlcChol *Helicobacter pylori*	C14	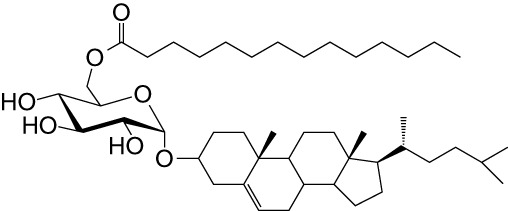	Strong; binds a small NKT cell subset (mo)	(53)
αGalDAG (GGL) *Borrelia burgdorferi*	*sn1*-C18:1 *sn2*-C16	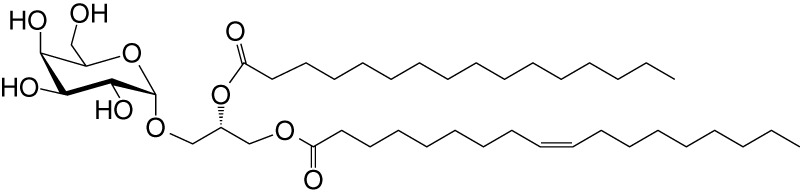	Weak (mo)-to-none (hu)	(54)
αGlcDAG (GGL) *Streptococcus pneumoniae*	*sn1*-C18:1 *sn2*-C16	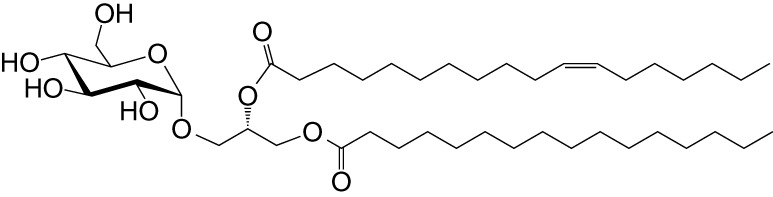	Weak	(55)
Self—mammalian cells				
αGalCer (GSL)	C18 C24:1	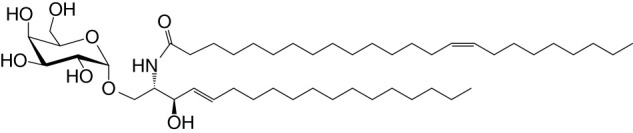	IFN-γ, IL-4	(56)
iGb3 (GSL)	C18- C24	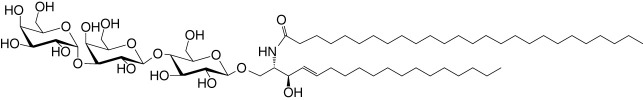	Weak (mo)-to-none (hu)	(57)

^1^Agel, agelasphin; Asp B, asperamide B; Chol, cholesterol; DAG, diacylglycerol; GalCer, galactosylceramide; GalUCer, galacturonosylceramide; GlcCer, glucosylceramide; *sn*, stereo nomenclature for glycerolipids; GGL, glycoglycerolipid; GSL, glycosphingolipid.

^2^sphingosine/phytosphingosine chain length indicated first and *N*-acyl chain length second,

^3^agonist strength based on Ref (58).

^4^relative potencies in comparison to αGalCer; mo, mouse; hu, human.

**Figure 3:**
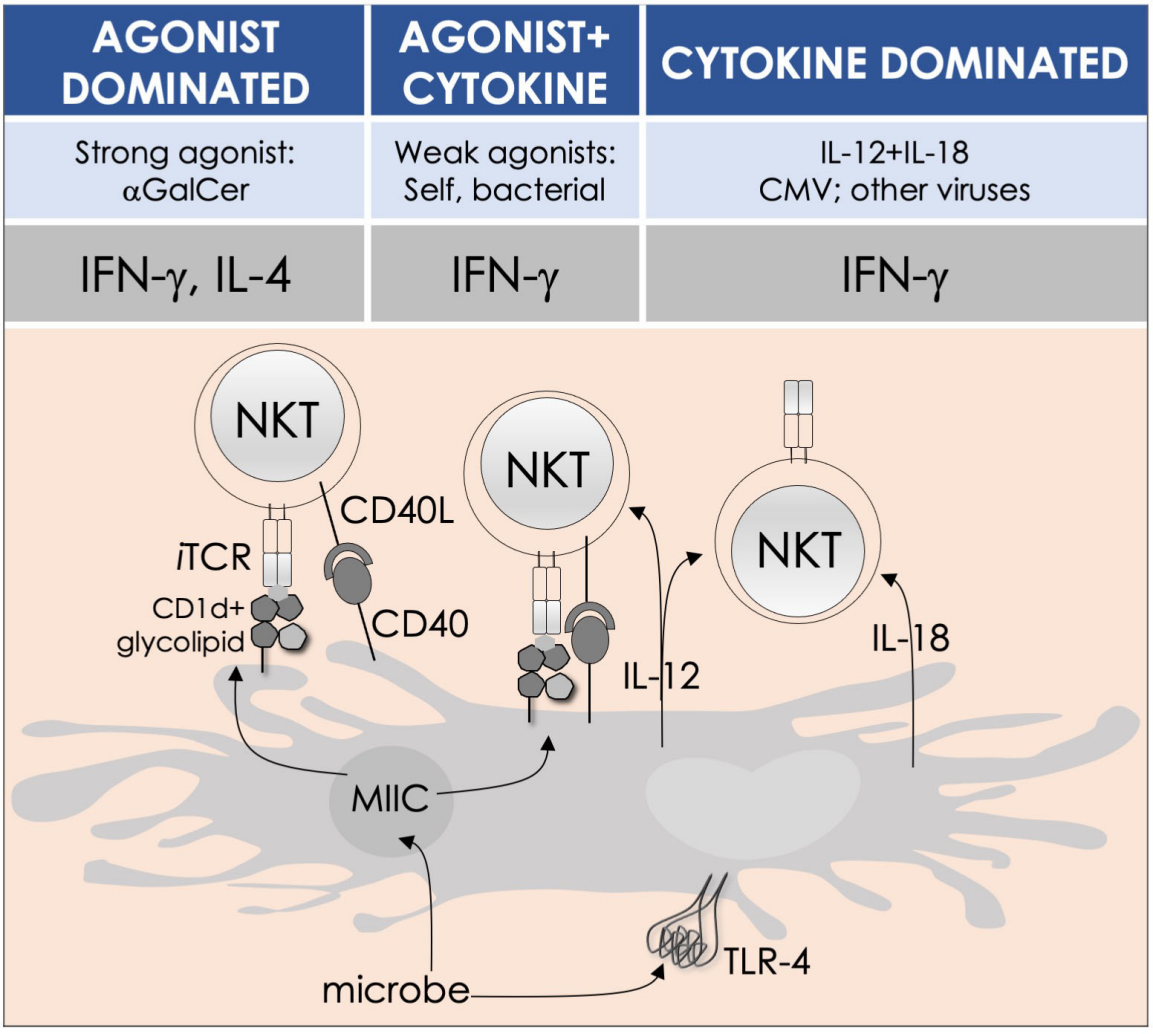
Modes of NKT and MAIT cell activation by microbes. Potent agonists—such as αGalCer, directly activate NKT cells, without the need for a second signal, in a TCR signaling–dominated fashion (left panel). Alternatively, microbes containing TLR ligands such as LPS activate NKT cells by inducing IL-12 production by DCs, which amplifies weak responses elicited upon the recognition of CD1d bound with self-glycolipids by the NKT cell TCR. Several endogenous lipid agonists have been identified and characterized (see **Table 1**). Some microbes—such as *Sphingomonas capsulata* and *Borrelia burgdorferi*—synthesize α-anomeric glycolipids for their cell walls. These glycolipids, when presented by CD1d, weakly activate NKT cells directly. In the presence of a second signal—generally a proinflammatory cytokine such as IL-12, such weak agonists strongly activate NKT cells (middle panel). By contrast, the mode of MAIT cell activation appears to be agonist concentration dependent: microbes that produce high levels of 5-OP-RU—a product of *ribD*-controlled catalytic activity, directly activate MAIT cells, while those that produce low levels of 5-OP-RU require a cytokine boost. Unlike conventional T cells, cytokines alone can activate both NKT and MAIT cells. Such cytokines, which include a combination of IL-12 and IL-18, activate NKT cells in a TCR-independent manner (right panel). This diagram renders the different strategies for NKT cell activation; they apply to MAIT cells as well. Similarities and differences, if any, are described in the text. Adapted from past reviews (35, 37, 38, 41) and works cited in the text.

By the last *fin-de-siècle*, the roles for NKT cells were implicated in steering immune responses to pathogens: to bacteria—*Salmonella choleraesuis*, *Listeria monocytogenes*, *Mycobacterium bovis*, and *M. tuberculosis*; to viruses—hepatitis B virus and lymphocytic choriomeningitis virus; to parasites—*Plasmodium* spp., *Leishmania major*, and *Schistosoma mansonii*; and to worms—*Nippostrongylus brasiliensis* [refs (63–80); see also **Supplemental Table 1**]. How NKT cells were activated by these pathogens was not understood. At that time, the only known NKT cell agonist was αGalCer (49, 81, 82). αGalCer (KRN7000) was isolated from the marine sponge—*Agelas mauritianus*, whose potent antitumor activity is mediated by NKT cells (47–49, 83) (see **Box 2**). In the ensuing two decades, much has been learnt about how NKT and MAIT cells control immune responses to infections with bacteria and viruses, many of which do not biosynthesize agonistic ligands. There are three distinct ways to activate NKT and MAIT cells (**Figure 3**): the first is termed TCR agonist–dependent direct activation. In this mode, the presentation of the agonist αGalCer by CD1d or 5-OP-RU by MR1 activates NKT or MAIT cells, respectively (**Tables 1** **–** **3**), to initiate their effector function/s (reviewed in ref (35). The second mode is termed TCR agonist–dependent and cytokine-assisted activation. Weak ligands—e.g., α-galacturonosylceramide (αGalUCer) biosynthesized by *Sphingomonas* spp (50, 51, 87, 111), αGalCer-like asperamide B by *Aspergillus fumigatus* (52), α-glycosyldiacylglycerols from *Borrelia burgdorferi* and *Streptococcus pneumoniae* (54, 55), or self αGalCer or isogloboside 3 (iGb3) induced by certain bacterial infections or sterile inflammation [ref (37, 56, 158–160); for structures, see **Table 1**]—that poorly activate NKT or MAIT cells require an immune push. That push is provided by inflammatory cytokines produced by the activation of DCs—e.g., IL-1β, IL-12, IL-18, or type I IFNs (96, 103, 116, 131, 155, 161). Hence, the context of infection can influence the activation of NKT and MAIT cells. The third mode of activation occurs in a manner independent of TCR stimulation but is reliant on cytokine/s alone. This mode of NKT and MAIT cell activation is termed TCR-independent inflammatory cytokine–induced activation. Bacteria that do not biosynthesize agonistic lipids but contain microbial pattern recognition receptor ligands such as lipopolysaccharide result in a TCR-independent inflammatory cytokine response from myeloid cells. These inflammatory cytokines can activate NKT cells. This mode of NKT and MAIT cell activation plays a protective role during infectious diseases, especially caused by virus infections (156, 157, 162–166).

**BOX 2 A tale of α-galactosylceramides and its biosynthesis.**

αGalCer/KRN7000 was first isolated from the marine sponge—*Agelas mauritianus*. As mammalian symbionts—e.g., *Bacteroides fragilis*, biosynthesize αGalCer-related compounds (26, 28, 84), it remains open whether the αGalCer was isolated from *A. mauritianus* or was derived from bacteria living in a symbiotic relationship with those sponges (85, 86). Bacteroidetes and α-Proteobacteria are the residents of sponges, members of which are known to biosynthesize α-anomeric glycosphingolipids that activate NKT cells (26, 28, 50, 51, 87). Of note, however, αGalCer was isolated from an *Agelas*-related marine sponge species—*Axinella corrugata* whose symbionts include α-Proteobacteria (88, 89). Nonetheless, current evidence suggests that the *A. corrugata* αGalCer was derived from the sponge itself and not its symbionts (88, 90). Resolving the source of αGalCer can yield insights into the biosynthesis of αGalCer in mammals (56). One possible route to the biosynthesis of αGalCer and αGlcCer might be the CGT1 (β-galactosylceramide synthase) and CGS (β-glucosylceramide synthase) themselves, which may have an α-linkage retention property. The two hexosylceramide synthases use α-linked uridyldiphosphate-charged sugar donors to form β-linked monohexosylceramides by catalyzing α to β mutarotation prior to the condensation reaction. The potential presence of αGlcCer/αGalCer in the absence of α-hexosylceramide synthase genes within mouse and human genomes poses a quandary, however (56, 91). Biochemical evidence suggests that hexosylceramide synthases may contain α-linkage retention activity, which retains the α-linkage of the charged sugar donor to generate α-linked monohexosylceramides (92–95). This α-anomer retaining activity may explain the synthesis of α-anomeric glycosphingolipids in sponges and mammals, and, potentially, in bacterial species discussed in the text that biosynthesize such lipids.

Once activated, NKT cells produce a variety of cytokines and chemokines that steer downstream innate and adaptive immune responses. This response includes type I, II, and III cytokines, which are secreted by NKT1, NKT2, and NKT17 cells, respectively. Corresponding MAIT1 and MAIT17 cells and attendant cytokine responses are similarly described. The three subsets emerge under the transcriptional activity of factors similar to those established in conventional CD4^+^ T cells (**Figures 1**, **2**). Broadly, akin to conventional CD4^+^ T cells, NKT and MAIT cells play roles in immunity to infections and tumors and in autoimmune and allergic reactions. These features of NKT and MAIT cells are reviewed in detail elsewhere (35, 38). In addition to the three NKT cell subsets, NKT10 cells—which secrete IL-10—play regulatory functions in conjunction with T regulatory cells. NKTfh cells—which provide cognate and noncognate help to conventional B cells to secrete antibodies—may control immunity to human pathogens such as *Borrelia hermsii*, *S. pneumoniae*, and *P. falciparum* (167–169). These features of NKT and MAIT cells are reviewed in detail elsewhere (35),

Human NKT cell responses are as diverse as the mouse NKT cells (170). Two functional subsets were recognized that were segregated by the lack of CD4 or CD8 coreceptor expression (NKT1) or by CD4 expression (NKT2). Human NKT1 cells produce IFN-γ and TNF-α and, when activated under the influence of inflammatory cytokines, upregulate NKG2D and perforin expression priming them for cytotoxic response against infected cells and cancer cells (171, 172). Akin to mouse, the human NKT2 subset, which produces IL-4 and IL-13 and their accumulation in the lungs, may underlie the pathology in chronic asthmatic patients (173). Activated human NKT cells also produce IL-17 (170), which may reflect the existence of an NKT17 subset in humans. Further, NKT17 and MAIT17 subsets are present in higher frequency when compared to NKT1 and MAIT1 subsets in liver perfusates, which produce IL-17 and IFN-γ, respectively (174). Human NKT and MAIT subsets have some semblance to mouse NKT and MAIT subsets, but further studies are necessary to understand how similar they are in the two species.

The evolutionary origins of NKT and MAIT cell subsets have not been traced yet. Both NKT and MAIT cells arose as eutherian innovations approximately 125 million years ago in an ancestor after the therian mammals split to metatherians and eutherians—the true placental mammals (35, 175, 176). Among mammals other than the mouse and human, the development and function of NKT cells in pigs—*Sus scrofa* (var. *domesticus*)—are intensely studied. Pig NKT cell subsets were recently described using the single-cell RNA sequencing analysis of more than 11,000 differentiating thymic NKT cells (177). The vast majority of porcine NKT thymocytes resemble mouse NKT2 cells. Surprisingly, these pig NKT2-like cells do not differentiate into NKT1 or NKT17 subsets. Instead, some develop into a population enriched for interferon-stimulated genes that simultaneously maintain an NKT2-like gene profile, as well as two very rare subsets, designated iNKT-swine (sw)1 and iNKT-sw2. iNKT-sw1 and iNKT-sw2 cells are most similar to two minor populations of innate-like CD8αα T cells present in pig thymocytes, sharing the expression of *FCGR3A*, *ZNF683*, *NKG7*, and MHC class II–encoding genes. They also downregulate tissue emigration genes, suggesting that both are long-term thymus residents. Similar thymus-resident populations of MAIT cells, γδ T cells, and CD8αα T cells have been described before and have been speculated to modulate thymocyte differentiation to respond to peripheral perturbations, such as infection (24, 174, 178, 179). Interestingly, iNKT-sw2 cells are enriched for *CD244* and *CXCR6*, which are upregulated on a newly discovered population of NKT cells found in mice and humans that are highly cytotoxic and protect mice from melanoma metastasis and influenza infection (180).

Although peripheral pig NKT cells can be stimulated nonspecifically to secrete IFN-γ and IL-17 (181, 182), thymus-resident pig NKT cells appear to produce little if any IFN-γ, IL-4, or IL-17 under steady-state conditions (177). One explanation for the surprisingly undifferentiated state of pig NKT thymocytes is that they emerge from the thymus in a functionally immature state and undergo further differentiation in the periphery. Since human NKT thymocytes do not also produce IFN-γ or IL-4 under steady-state conditions, it is possible that the diversity of NKT thymocyte subsets observed in mice is unusual and that it is more normal for species with the NKT-CD1d system to express fewer and/or less differentiated NKT thymocytes.

In comparison to NKT cells, relatively little is known about porcine MAIT cells. However, MAIT cell TRAV1-TRAJ33 TCRα sequences have been cloned from pig blood and tissues and found to pair with a limited number of TCR β-chains (183). It was further shown that pig MAIT cells can be CD4 ^pos^ CD8 ^pos^ , CD4 ^pos^ CD8 ^neg^ , and CD4 ^neg^ CD8 ^pos^ T cells and express transcripts for the MAIT cell–associated surface molecules IL-18Rα, IL-7Rα, CCR9, CCR5, and/or CXCR6 and the transcription factors PLZF and T-bet or RORγt.

Collectively, current evidence indicate that pig NKT and MAIT cells have characteristics similar to their human and mouse counterparts. Nonetheless, several key lineage-defining differences in mouse and pig NKT cell subsets point toward the acquisition of species-specific innate/innate-like T cell adaptations, perhaps for different pathogens or may reflect the different niches in which the two species evolved and the symbiotic microbes they live with. Hence, the species-specific developmental aspects should be considered, especially in the light of ecology and evolution, when assessing the suitability of mice and pigs as biomedical models for innate/innate-like T cell research.

## The hygiene hypothesis: yes, you may pick your nose and eat it

This subtitle was motivated by a burgeoning field of rhinotillexis—yes, nose picking, a new area of scientific enquiry. Beneath this otherwise aversive and socially inept and unacceptable behavior, yet innate to primates, may lie a means to the periodic reinforcement of disease tolerance [see **Box 3**; ref (184)].

**BOX 3 Rhinotillexis—a new, burgeoning field of scientific enquiry.**

It is so new and burgeoning that the National Public Radio felt compelled to interview Dr. Anne-Claire Fabre—a pioneer in the field at the Naturhistorisches Museum in Bern, Switzerland, on the matter (npr.org/2022/11/15/1136423436/researchers-dig-into-why-nose-picking-is-a-common-behavior). It is so new that the word rhinotillexis is neither in the Oxford English Dictionary nor the Merriam-Webster American English Dictionary yet but has appeared in Wikipedia, the free encyclopedia, however. Unless careful, excessive rhinotillexis may cause self-induced ethmoidectomy, especially if one suffers from rhinotillexomania (see en.wikipedia.org/wiki/Nose-picking). Rhinotillexis is not peculiar of repulsive men or their man cubs, but it is a primate thing [(184) and references therein]. Self-vaccination, per oral distribution of nasal microflora, and dental hygiene are a few proposed immunologic attributes of rhinotillexis (185–187).

The pervasive presence of microbes, flourishing at every nook and cranny of the earth and on the surfaces and the insides of metazoans, make them a formidable friend and foe. Hence, on being birthed unto a dirty world, to gain fitness, metazoans found ways to befriend and tame microbes, especially the beneficial, and ward off unfriendly ones over eons of evolution. Symbiosis emerged, lending to fitness in both directions—in the metazoan hosts and their microbial partners. So much so, symbiosis has led to the coevolution of the hosts with their microbiota, or vice versa, to the point of codependence, wherein the immune system evolved to manage the microbial consortium from going ‘wild’ and, reciprocally, the diversity of the consortium and its biosynthetic products control the immune system from going ‘rouge’. Thus, the hygiene hypothesis postulates that early life exposure to a full range of diverse microbes (and worms) promotes the development and maturation of an immune system—which reacts in a balanced measure to prevent disease whether incited by external (infections and allergens) or internal (autoinflammation) agencies of inflammation (188).

For example, under sterile, germ-free conditions, the immune system of the laboratory mouse develops and matures poorly, rendering them susceptible to infectious diseases and autoimmune disorders such as colitis (189–196). Conversely, the equilibration of the gut microbiome of the laboratory mouse to that of the ‘dirty’ pet store mouse by cohousing the two, altered, in the former, the immune cell composition at the barrier sites, resistance to infection, and T-cell differentiation in response to virus infection (197). A similar equilibration of the gut microbiome of a laboratory mouse raised under germ-free conditions by the transfer of the gut microbiota from a feral relative of the laboratory mouse and its maintenance over several generations by breeding increased disease tolerance and fitness. Inflammatory responses in such mice to a lethal influenza virus challenge was highly tempered and so was mutagen- and inflammation-induced tumorigenesis (198). All of these altered immune features acquired by the laboratory mouse reflected those of the pet store or feral mouse and those of the adult human (197, 198). The ability to approximate the human immune system in the laboratory mouse by the transfer of the microbiome indigenous of a feral mouse may facilitate and enhance preclinical vaccine development and testing (198–201). Furthermore, the role of the microbiota in the maturation of T cells may explain the intriguing finding that, at steady state—in the absence of an infection—DC emigrees from the barrier epithelium of nonlymphoid tissues stochastically prime and program resting, naïve CD8^+^ T cells within the local draining lymph nodes for tissue residency (202).

After development in the thymus, NKT and MAIT cells emigrate and home to lymphoid and nonlymphoid tissues, presumably to patrol and maintain the integrity of the tissue borders. The NKT and MAIT cell content at these borders varies by tissues and the mouse strain. Their tissue distribution and functions are best studied in the mouse; only a bit is known of their distribution in the human body (24, 172, 203–205). In mice, thymic NKT cell development, after commitment to this lineage and positive selection, progresses from stage 0 to stage 1 to stage 3—the mature NK1.1 ^pos^ NKT cells, known to consist largely of NKT1 cells. Of these, CD24 ^neg^ CCR7 ^pos^ stage 1/2 NKT cells emigrate from the thymus and seed both the lymphoid and non-lymphoid tissues, where they undergo further maturation, largely driven by the local cytokine milieu (206–208), and perhaps the microbiota.

Verily, the early life exposure of NKT and MAIT cells to the host microbiota has profound, lifelong effect/s on these innate-like lymphocytes (25, 27, 161). Their development itself is dependent on positive selection by agonistic ligands—αGalCer in the case of NKT cells and 5-OP-RU in the case of MAIT cells [reviewed in refs (44, 203), and references therein]. The origins of these agonists are less clearly defined. Because CD4 ^pos^ CD8 ^pos^ thymocytes activate Vα14i NKT cell hybridomas, it is thought that an NKT cell agonist/s may be of self origin. Thus, β-galactosylceramide synthase (CGT)-deficient thymocytes foster NKT cell development; hence, βGalCer or its derivatives are less likely the thymic NKT cell agonist. Conversely, β-glucosylceramide synthase (CGT-1)–deficient thymocytes poorly activate Vα14i NKT cell hybridomas and conditional CGT1-deficient thymocytes to not promote NKT cell development (57, 209). As βGlcCer itself does not activate Vα14i NKT cell hybridomas, a βGlcCer derivative—iGb3 or a self αGlcCer (**Table 1**)—is a potential NKT cell–activating self-agonist. While iGb3 synthase deficiency does not alter NKT cell development and function and no known mammalian enzyme/s synthesize α-anomeric glucosylceramide or galactosylceramide, how these agonists are biosynthesized is unclear [see **Box 2** for details, see ref (35)]. Alternatively, as several gut symbionts common to many mammals biosynthesize α-anomeric glycosylceramides, their transport by lipid transfer proteins such as apolipoprotein E (210) could potentially deliver the agonist/s to the thymus. This is less likely because NKT cells develop in germ-free mice, but they are not without defects (25, 27, 211).

In a similar vein, mammalian cells do not biosynthesize vitamin B2, whose precursor is a precursor to the MAIT cell agonist 5-OP-RU (33, 59, 147, 212), but rather acquire it from symbionts (161, 203, 213). Consequently, MAIT cells develop poorly in germ-free mice bred under sterile conditions (205, 214). By contrast, NKT cells develop in such mice as noted above. It appears as though NKT cells and MAIT cells compete for niche such that, mice, which have more NKT cells than humans, have a low frequency of MAIT cells. Reciprocally, humans have a high frequency of MAIT cells but are low in NKT cell frequency (205, 214).

NKT cell numbers in the intestinal mucosa are controlled by the neonatal colonization of bacterial symbionts. NKT cells accumulate in significant numbers within the intestinal mucosa, lungs, and liver but not the thymus or spleen of germ-free mice (25, 27). The increased NKT cell number observed in germ-free mouse intestinal mucosa perhaps owes to increased levels of CXCL16—the ligand of CXCR6, the levels of which are controlled by the gut microbiota (25, 215). Moreover, NKT cells developing in germ-free mice do not mature and are hyporesponsive to the glycolipid agonist αGalCer (27). Colonization with NKT cell agonist–bearing bacteria—e.g., *Sphingomonas yanoikuyae*, during early life but not in adulthood restored NKT cell maturation and normoresponsiveness to αGalCer (27). Nevertheless, αGalCer compounds synthesized by different bacterial symbionts—e.g., *Bacteriodes fragilis* and *S. yanoikuyae* (see **Table 1**), appear to exert differential effects on developing NKT cells (26, 28, 84); why this is awaits resolution.

Early-life microbial ecology has implications for health. Thus, consistent with increased NKT cell frequency in the gut and lungs, germ-free mice are overly sensitive to oxazolone/dextran sodium sulfate–induced inflammatory colitis and airway hypersensitivity (25, 27, 215). This disease phenotype is reversed by early-life exposure to *B. fragilis–*derived glycosphingolipid(s) (28). Whether the normal development and functions of human NKT cells require interactions with the gut microbiota awaits discovery. So also, whether the microbiota—known to vary between individuals of different genetic, ethnic, and geographic backgrounds (216)—controls human peripheral NKT cell frequency, which varies tremendously between individuals—from undetectable to 5%—remains unknown.

Unlike the gut, which hosts swarms of thousands of microbial species, it is generally assumed that the internal organs not exposed to the outside—such as the liver, heart, and brain—are sterile, devoid of resident microbes. Counter to this assumption, a recent study found mouse and human liver hosts its own, unique microbial consortium distinct from the gut as it was enriched in Proteobacteria (217). This microbiome was seeded from the gut microbiota in a selective manner that depended on the sex of the mouse and the local environment. Moreover, the local immune response was dependent on the liver microbiome, which was influenced by *Bacteroidetes* species. The hepatic microbiome controlled antigen-presenting cell maturation and adaptive immunity through the mediation of NKT cells (217). *Bacteroidetes* species biosynthesize αGalCer (26, 28, 84), which activate NKT cells to secrete CCL5 chemokine, in turn, recruiting immune cells to the liver and their activation, expansion, and function (217). Hence, local tissue microbiomes influence local immunity in an NKT cell—dependent mechanism.

NKT cell homeostasis described above requires intestinal microbial lipid presentation by CD11c^+^ DCs and macrophages (218). Reciprocally, NKT cells appear to control the bacterial composition of the gut microbiota. Consequently, dysbiosis and disruption in intestinal homeostasis ensue in mice deficient in NKT cells—CD1d^-/-^ (218–221) or Jα18^-/-^ (222–224) mice —or mice that lack CD1d expression by DCs, which thereby are unable to present intestinal lipids to activate local (218), intestinal mucosal NKT cells. This dysbiosis and altered intestinal homeostasis are consistent with alterations in the IgA repertoire (223, 225) and the induction and function of regulatory T cells within the gut (192, 222), which are observed in these mice as well (218, 223).

By contrast to the above reports, a recent study found that there are no differences in the composition of the gut microbial consortium in CD1d^−/−^ mice (226). Similarly, no differences were observed in the consortium in Vα14 transgenic mice, which carry high numbers of NKT cells—largely the IL-4 producing NKT2 subset (227, 228). While NKT cell activation by peroral delivery of αGalCer minimally, yet consistently, altered the diversity of the consortium, this effect was only transient. However, the shift in microbiota composition was comparable to the natural drift found in the colony. Critically, this report noted that the natural drift in the microbial composition of individual vivarium over time and, perhaps, the differences in the microbial composition between vivaria, but not NKT cells, had significant influence on the composition of the mouse gut microbial consortium even at steady state (226). Because this is a report from a single center, whether mouse and human NKT cells have an impact on the microbial consortium of the gut will require a concerted, multicenter study.

Mouse and human skin abound with MAIT cells. MAIT cell frequency varies between individuals (229). MAIT cell frequency is similar in genetically identical mice housed in the same cage but varied between those housed in distinct cages. This suggested that the microbiota may have a role in determining the frequency. Studies in germ-free mice revealed that MAIT cells depended on early-life exposure to gut microbial consortium (45, 161, 203, 213). Hence, germ-free mice failed to develop MAIT cells that localize to barrier tissues—such as the skin, when exposed to microbes later in life.

The development of mouse MAIT cells in the thymus is dependent on the presentation of a by-product of riboflavin biosynthesis—5-OP-RU (33, 44, 59, 147, 203, 212). Even though flavonoids are essential, mammalian cells are riboflavin auxotrophs. They depend on external sources of riboflavin, which is biosynthesized by several bacteria and fungi—both symbionts and pathobionts, as well as plants. The microbial origin of riboflavin and biosynthetic metabolites explains the intimate dependence of MAIT cell development on the gut microbiota. 5-OP-RU is biosynthesized in a *ribD*-dependent manner by the gut, and potentially the skin as well, transported to the thymus, and made available to MR1-expressing cells for assembly and display at the cell surface (213). The mechanism by which 5-OP-RU is transported to the thymus and how cells capture it to make available in the ER lumen for assembly with MR1 are poorly, if at all, understood (203).

Thymic MAIT cell emigrees home to barrier tissues. Their numbers at the barrier tissues depend on the local concentration of microbial derivatives, which is emulated by the painting of skin with varying concentrations of 5-OP-RU (161, 213). In the skin, they surveil the dermal—epidermal interface. Cutaneous-resident cells are the MAIT17 subset; their homeostasis is IL-23 dependent, and they respond to skin commensals upon MR1-ligand recognition in an IL-1- and IL-18-dependent manner. These MAIT17 cells are genetically programmed for tissue repair and, hence, contribute to normal skin physiology (161). Given the intimacies of NKT and MAIT cells with the symbiotic consortium, one might wonder what roles innate-like effector lymphocytes might have in precipitating *erythema toxicum neonatorum*—which is perhaps an innate immune response to skin microbiont/s that may have penetrated the newborn infant (230).

When van Leeuwenhoek peered down his microscope, curious what might live on his teeth, and perhaps his gums, little did he know he would find many ‘little animals’. In his *Letter 39* to the Royal Society, he claimed,

“*For my part I judge, from myself (howbeit I clean my mouth …), that all people living in our United Netherlands are not as many as the living animals that I carry in my own mouth this very day: for I noticed one of my back teeth, up against the gum, was coated with the said matter for about the width of a horse-hair, where, to all appearance, it had not been scoured by the salt for a few days; and there were such an enormous number of living animalcules here, that I imagined I could see a good number of ‘em in a quantity of this material that was no bigger than a hundredth part of a sand-grain*” (from a collection of surviving van Leeuwenhoek letters, translated and compiled in ref (6). [see letter 39: *Phil. Trans*. XIV (231) 568, 1684)].

What those ‘little animals’ or ‘animalcules’ on man’s teeth meant remained cloaked for over two centuries. Elie Metchnikoff had a hunch to which, later in his career and life, he laid, to an obsession, much attention to prolong his life, in futility notwithstanding (232). The foregoing advances, which awaited next-generation ‘omics’ technologies and platforms, vindicates Metchnikoff’s hunch on beneficial and harmful gut microbes and lends support to the physiologic functions of early-life exposure to a diverse array of microbes—and, hence, the hygiene hypothesis.

## *Kämpfe únd schláchten* of natural killer T and mucosal-associated invariant T cells with pathogens

NKT cells and MAIT cells perform specialized roles during infections to confer immunity to the host as they struggle (kampf) with and battle (schlacht) pathogens (see **Tables 2**, **3** and **Supplementary Tables 1**, **2**). While both possess the phenotype of activated T cells, their induction differs from conventional T cells in that they can be triggered during pathogen infections through invariant receptors and cytokine signals in much the same fashion as innate cells. This results in the rapid secretion of multiple cytokines that are released with similar kinetics to innate cell-derived cytokines—i.e., minutes to hours after stimulation. Accordingly, NKT and MAIT cells can influence the behavior of cells in the innate branch of the immune response while also shaping downstream adaptive immune responses. Over the past decades, it has become clear that the innate properties of NKT and MAIT cells are shared by a wide variety of MHC class I–like restricted innate-like αβ T cells with invariant TCRs that are widespread among jawed vertebrates [reviewed in Ref (233)]. These types of lymphocytes are specialized to allow the recognition of common or particular pathogens with relatively few T cells (231). A good example is *Xenopus laevis* (African clawed frog) tadpoles, which are able to survive in antigen-rich waters using 15,000–20,000 T cells exhibiting limited TCR diversity (234).

**Table 2:** Role of NKT cells in microbial infection and immunity.

Microbe	Activation mechanism/s (antigen)^1^	NKT cell role^2^	Model	Infection route	Reference(s)
Gram-positive bacteria					
*S. pneumoniae*	CD1d-dependent self and nonself (αGalDAG) + IL-12	Protective	Jα18^-/-^, CD1d^-/-^	i.n., i.t.	(55, 96, 97)
*S. aureus*	Non-self (lysyl-PG)	Not protective	Jα18^-/-^, CD1d^-/-^	i.v.	(98, 99)
*L. monocytogenes*	Self + IL-12	Protective Detrimental	CD1d^-/-^	i.v.	(99–101)
Gram-negative bacteria					
*P. aeruginosa*	CD1d-dependent (unknown)	Protective	CD1d^-/-^	i.n.	(102)
		Not protective	Jα18^-/-^, CD1d^-/-^	i.t.	
*S. typhimurium*	CD1d-dependent self (iGb3)	Not protective	CD1d^-/-^	p.o.	(51, 99, 103, 104)
*H. pylori*	CD1d-dependent nonself (αCgT)	Protective	Jα18^-/-^	p.o.	(105)
*C. trachomatis (muridarum)*	CD1d-dependent nonself (GLXA)	Detrimental Not protective	CD1d^-/-^	i.n. intravaginal	(106–108)
*C. pneumonia*	CD1d-dependent self and nonself (unknown)	Protective	Jα18^-/-^, CD1d^-/-^	i.n.	(109)
*L. pnemophilla*	Cytokine dependent, IL-12	Detrimental	Jα18^-/-^	i.t.	(106–108)
*Francisella tularensis* subspp. *tularensis SchuS4*	CD1d dependent (unknown)	Detrimental	CD1d^-/-4^	i.n.	(110)
*Ft* subspp. *holarctica* live vaccine strain	CD1d dependent (unknown)	Detrimental	CD1d^-/-^	i.n.	(110)
*F. novicida*	CD1d dependent (unknown)	Not protective	CD1d^-/-^	s.c., i.d.	(110)
α-Proteobacteria					
*Sphingomonas* spp.	CD1d dependent nonself (αGlcACer) + IL-12	Protective (low dose) Detrimental (high dose)	Jα18^-/-^, CD1d^-/-^	i.v.	(50, 51, 96, 111)
*N. aromaticivorans*	CD1d-dependent nonself (αGalUCer)	Primary biliary cirrhosis	CD1d^-/-^	i.v.	(112)
**Spirochetes**					
*B. burgdorferi*	CD1d-dependent, nonself (αGalDAG) + IL-12	Protective	^4^CD1d^-/-^	i.d.	(54, 96, 113, 114)
Mycobacteria					
*M. tuberculosis*	CD1d-dependent self	Not protective Protective^3^	CD1d^-/-^ Cell transfer	i.v. aerosol	(72, 115)
Fungi					
*A. fumigatus*	CD1d-dependent non-self (asperamide-B) and self + IL-12	Detrimental (AHR)^3^ Protective (early)	CD1d^-/-^	i.n. i.t.	(52, 116)
*C. neoformans*	CD1d-dependent self	Protective	CD1d^-/-^	i.t.	(117)
Parasites					
*P. berghei*	ND	Detrimental	CD1d^-/-^	i.d.	(118)
*P. yoelii*	CD1d dependent	Protective	CD1d^-/-^	i.v.	(119)
*T. gondii*	ND	Protective Detrimental^3^	Jα18^-/-^, CD1d^-/-^ Jα18^-/-^, CD1d^-/-^; Vα14^tg^	p.o.	(120, 121)
*L. donovani*	CD1d dependent, lipophosphoglycan	Protective	CD1d^-/-^	i.v.	(122)
*E. histolytica*	CD1d dependent, foreign antigen (EhLPPG)	Protective	CD1d^-/-^	i.h.	(123)
Viruses					
HSV-1	CD1d dependent, nonself (glycoprotein B and US3)	Protective Not protective^3^	Jα18^-/-^, CD1d^-/-^	Scarification	(124–126)
HSV-2	ND	Protective	CD1d^-/-^	Intravaginal	(127)
Sendai virus	ND	Detrimental	Jα18^-/-^, CD1d^-/-^	i.n.	(128)
RSV	CD1d dependent, self	Protective	CD1d^-/-^	i.n.	(129, 130)
Influenza virus H1N1 and H3N2	ND	Protective	Jα18^-/-^, CD1d^-/-^	i.n.	(131–135)
HBV	ND	Protective	Jα18^-/-^, CD1d^-/-^	i.v.	(136)

^1^see **Table 1** for the structures of NKT cell agonists.

^2^differential outcomes in the different studies may have arisen from the use of different microbial/parasite strains.

^3^the outcome of infection in Jα18^-/-^ mouse model may require additional validation as the deletion of this TRAJ gene segment by homologous recombination had resulted in the deletion of additional TRAJ gene segments, including TRAJ33—the gene segment essential for the construction of MAIT cell TCR α-chain; additional TRAJ gene segment losses severely constricted the TCR repertoire of conventional T cells as well [see ref (137)].

^4^BALB/c background of mouse strains used in these studies; others were in C57BL/6 background.

AHR, airway hyperreactivity; GLXA, chlamydial glycolipid exoantigen; ND, not determined; i.d., intradermal; i.h., intrahepatic; i.n., intranasal; i.p., intraperitoneal; i.v., intravenous; i.t., intratracheal; p.o., per oral; s.c. subcutaneous.

**Table 3:** Role of mucosal-associated invariant T cells in microbial infection and immunity.

Microbe	Activation mechanism/s	MAIT cell role^1^	Model	Infection route	Reference(s)
Gram-positive bacteria						
*C. difficile*	MR1 and cytokine dependent	Detrimental	Human PBMC	*in vitro*	(138)
*S. pneumoniae*	MR1 dependent, SAgs	Detrimental	C57BL/6, CAST : EiJ	*in vitro*	(139)
	MR1 dependent, Spn polysaccharide	Protective	Human PBMCs	*in vitro*	(140)
	MR1 (SAgs) and cytokine dependent IL-12 and IL-18	Detrimental	Human PBMCs	*in vitro*	(141)
*S. aureus*	MR1 dependent, SAgs	Detrimental	C57BL/6, CAST : EiJ	*in vitro*	(139)
Gram-negative bacteria						
*K. pneumoniae*	ND	Protective	MR1^-/-^	i.p.	(142)
*P. aeruginosa*	ND	Protective	Human PBMCs	*in vitro*	(143)
*L. longbeachae*	MR1 dependent	Protective	MR1^-/-^	i.n.	(144)
*H. pylori*	MR1 dependent	Detrimental	MR1^-/-^	p.o.	(145, 146)
*E. coli*	MR1 dependent	Protective	Vα19^tg^, MR1^-/-^	i.p., i.v.	(147)
*S. enterica* serovar *Typhi*	MR1 dependent	Detrimental	Human PBMCs	*in vitro*	(148)
*S. enterica* serovar *paratyphi A*	MR1 dependent	Protective	Human PBMCs	*in vitro*	(149)
*S. typhimurium*	MR1 dependent	Protective	Human PBMCs	*in vitro*	(33)
*F. tularensis* subspp*. holarctica LVS*	ND	Protective	MR1^-/-^	i.v.	(150)
	MR1- and cytokine- dependent IL-12p40	Protective	MR1^-/-^	i.n.	(151)
Mycobacteria						
*M. abscessus*	MR1 dependent	Protective	Vα19^tg^, MR1^-/-^	i.p., i.v.	(147)
*M. tuberculosis*	MR1 dependent, riboflavin derivatives	Protective	C57BL/6, Cast;EiJ	i.n.	(152)
Viruses						
Dengue virus	Cytokine dependent: IL-12 and IL-18	Protective	Human PBMCs	*in vitro*	(153)
Zika virus	Cytokine dependent: IL-12 and IL-18	Protective	Human PBMCs	*in vitro*	(153)
HIV-1	Cytokine dependent: IL-12 and IL-18	Protective	Human PBMCs	*in vitro*	(154)
Influenza A	MR1 and cytokine dependent	Protective	Human PBMCs and LDMCs	*in vitro*	(155)
	Cytokine dependent: IL-18	Protective	Human PBMCs	*in vitro*	(156)
Influenza virus H1N1	Cytokine dependent: IL-12 and IL-18	Protective	MR1^-/-^	i.n.	(157)

^1^differential outcomes in the different studies may have arisen from the use of different microbial/parasite strains.

ND, not determined; i.n., intranasal; i.p., intraperitoneal; i.v., intravenous; p.o., per oral.

As regard the role of NKT cells in immunity, mice deficient in CD1d or TRAJ18 that lack invariant NKT cells have shown that these cells play nonredundant roles in several models of infectious disease (235); NKT cell–deficient mice are more susceptible to several bacteria species (**Table 2** and **Supplemental Table 1**), including *S. pneumoniae* (97, 236), *Borrelia burgdorferi* (113), *Sphingomonas* spp. (50, 51), *Pseudomonas* spp. (102), *Chlamydia pneumoniae* (109), and *M. tuberculosis* (73). They also exhibit greater susceptibility to fungal infections with *Cryptococcus neoformans* (117) and *Aspergillus fumigatus* (116); viral infections with herpes simplex virus (124, 237), hepatitis B virus (80, 136), and influenza A virus (131, 132, 238); and protozoan parasite infections with *Plasmodium* spp (76) and *L. donovani* (239). A wide array of microbes and microbial products can stimulate NKT cells, either by direct TCR activation, cytokine-mediated activation, or a combination of both and induce them to express activation markers and cytokines, which have diverse effects on other immune cells and the course of an infection (see **Tables 1**, **2** and references therein). Indeed, microbially activated NKT cells typically secrete a narrower range of cytokines than αGalCer-stimulated NKT cells, which are usually predominated by IFN-γ. This is consistent with the paradigm that the microbial activation of NKT cells is mediated, to a large extent, through innate cytokines such as IL-12 and IL-18, with weak or no TCR stimulation (240). In some infections, NKT17 cells play a significant role. NKT17 cells in a granulocyte–monocyte colony-stimulating factor (CSF2)–dependent manner plays a protective role against *S. pneumoniae* infection of mouse lungs (236). While *Csf2*-deficient NKT cells are impaired in αGalCer-induced cytokine secretion and the transactivation of downstream innate and adaptive immune responses (241), anti-CSF2 blocking experiments confirm the role of NKT17 cell–derived CSF2 in immunity against *S. pneumoniae* (236). Moreover, NKT cells activated by microbes do not usually undergo systemic expansion *in vivo* even when they contain NKT cell antigens. However, NKT cells have been found to congregate at the sites of infection in mice infected with lymphocytic choriomeningitis virus (79), malaria parasites (119), and *C. neoformans* (117). They have also been shown to expand in the lungs and draining lymph nodes of pigs infected with influenza and in the peripheral blood, draining lymph nodes, and lungs of pigs infected with African swine fever virus (182). An intriguing aspect of NKT cell biology is that these cells are programmed to undergo apoptosis and/or become functionally anergic after stimulation (242–244). This reduces the risk of a cytokine storm or chronic inflammation arising from the large efflux of proinflammatory cytokines that activated NKT cells produce. Usually, the degree of NKT cell deletion/dysfunction corresponds with the strength of activation, with some microbes such as the lymphocytic choriomeningitis virus capable of rendering NKT cells anergic for up to 3 months after infection (79, 245). Nevertheless, the overactivation of NKT cells does occur in some mouse models of infection, especially in tissues where NKT cells are found at high concentrations, such as the liver in mice (80, 246, 247).

Among the lessons learnt from studying NKT cells in mice is that genetic background can strongly influence the immunomodulatory activities of NKT cells. For example, the same αGalCer analog treatment protocols cause divergent effects on disease between different mouse strains in the mouse models of autoimmune diabetes (248), experimental autoimmune encephalomyelitis (249), collagen-induced arthritis (250, 251), and systemic lupus erythematosus (252). Such differing outcomes are probably related to the diverse concentrations and functional phenotypes of NKT cells that exist among inbred mouse strains. For instance, in a survey of 38 inbred mouse strains, NKT cells as a percentage of αβ T cells ranged from 3.2% to 0.01% in peripheral blood, 4.12% to 0.02% in the spleen, and 9.39% to 0.02% in the thymus (253). The proportion of CD4^+^ to CD4^-^CD8^-^ double- negative NKT cells showed similar profound strain variation. Functional differences have been ascribed to these subsets, with the CD4^+^ subset exerting immunological tolerance in several disease models.

Humans present comparable levels of heterogeneity in NKT cell frequency and cytokine secretion profiles (171, 172, 254–258), which may result in distinct NKT cell responses to microbial infections that vary between individuals. However, whether NKT cells play nonredundant roles in human infectious diseases is largely unknown. Infection with the human immunodeficiency virus, dengue virus, and *M. tuberculosis* have been linked to reduced NKT cell responses to subsequent αGalCer stimulation (259–261). While these results suggest that at least some of the findings from mouse NKT cell studies apply to human infections, there is little evidence to indicate that humans with unusually high or low NKT cell concentrations or effector responses have altered susceptibility to microbial infections. Moreover, assessing this relationship is complicated by the fact that circulating NKT cells are often a poor reflection of NKT cells in organs and tissues (253, 254). In due course, questions about the translatability of mouse model studies may be partly addressed using CD1d knockout pigs as pig and human immune systems share many similarities, and pigs can be infected with a wide range of human pathogens (262–266).

MAIT cells are activated by microbial species that have an intact riboflavin pathway (**Table 3**). Accordingly, mice deficient in MAIT cells have an impaired ability to clear 5-OP-RU-producing bacteria, such as *Francisella tularensis* (151, 267), *M. bovis* bacillus *Calmette*-Guérin (268), *M. abscesses* (147), and *Legionella longbeachae* (144). Furthermore, TRAV1-TRAJ33 TCR-transgenic mice that express high concentrations of MAIT cells are more resistant to disease in a mouse model of *M. tuberculosis* infection (147). The mechanisms underlying MAIT cell antimicrobial immunity are not fully understood (see **Supplemental Table 2**). However, MAIT cells can lyse infected cells through perforin and granzymes (269, 270). They also secrete a variety of effector cytokines, such as IFN-γ, TNF-α, GM-CSF, and IL-17, which potentiate bacterial killing through myeloid cell activation (44, 196, 203, 205, 271, 272).

In addition to TCR-mediated activation, MAIT cells can respond to microbial infections through a variety of cytokine receptors that these cells express, including receptors for IL-1, IL-7, IL-12, IL-15, IL-18, and IL-23 (203, 271). This capacity for TCR-independent stimulation enables MAIT cells to participate in immune responses against viruses that do not produce 5-A-RU derivatives. For instance, in a mouse model of lethal influenza virus infection, MR1-deficient mice had a significantly higher mortality rate than MR1-intact mice (157). Similar results have been reported for both CD1d and TRAJ18 knockout mice demonstrating that NKT cells also play a nonredundant role in influenza virus infections (131, 132, 238). However, while NKT cells were found to be important for inhibiting virus replication, MR1-deficient mice had a similar virus load to MR1-intact mice. Moreover, TCR-dependent stimulation was found to be indispensable and dispensable for NKT cells and MAIT cells, respectively, to control influenza virus infections (132, 157). These results suggest that there exists significant overlap as well as cell type–specific differences in the antiviral activity of NKT cells and MAIT cells.

The role of MAIT cells in human antimicrobial responses remains largely uncertain. However, their high abundance in humans suggests that they may play a more prominent role in host defense and tissue homeostasis than they do in mice. MAIT cell deficiencies have not been directly associated with susceptibility to a particular pathogen in humans. Nevertheless, the frequency of MAIT cells has been found to decrease in the blood of humans infected with various types of bacteria. In some cases, this was accompanied by an increase in MAIT cell frequency at the site of infection (203, 272), suggesting that circulating MAIT cells migrate from circulation to the infection site.

In addition to their contribution to antimicrobial immunity, MAIT cells play a role in wound healing, including repairing host tissues damaged by immune cells during pathogen clearance (203, 272). Activated MAIT cells express a variety of tissue repair factors, including TGF-α, amphiregulin, vascular endothelial growth factor A, IL-5, IL-13, and IL-22 (155, 273, 274). MAIT cells in barrier tissues of the lung and skin are particularly enriched for tissue repair genes, and MAIT cell–mediated wound healing has been demonstrated in punch biopsy and *Staphylococcus epidermis* infection models of skin damage (161). Together, these findings indicate that MAIT cells play Janus-like opposing roles during infection, on the one hand promoting cytotoxic and proinflammatory responses that destroy infected cells while also restoring tissue integrity after the resolution of the infection.

## Stymied by microbial stealth

Unsurprisingly, pathogens have devised ways to stymie CD1d-restricted antigen presentation. Most evade intracellular CD1d trafficking. For example, the modulator of immune recognition (MIR)-1 and MIR-2 proteins of Kaposi sarcoma–associated herpesvirus (KSHV) are ubiquitin ligases. The two KSHV proteins ubiquitinylate the cytoplasmic tail of human CD1d, forcing the endocytosis of surface CD1d and, thereby, reducing cell-surface CD1d expression (275). The human immunodeficiency virus 1-encoded Nef protein mirrors the effects of MIR-1 and MIR-2 proteins to reduce CD1d expression perhaps by increased endocytosis coupled with the inhibition of the return transport of CD1d to the cell surface (276, 277). Similarly, in herpes simplex virus 1 (HSV-1)–infected cells, CD1d molecules accumulate in the MHC class II–enriched compartment due to a defect in CD1d recycling from endosomal compartments back to the cell surface (278). HSV-1 also inhibits the upregulation of cell surface MR1 *via* the US3 gene product to evade MAIT cell recognition (279). Vaccinia virus and vesicular stomatitis virus also abrogate CD1d antigen presentation, likely by impeding the intracellular trafficking of CD1d molecules induced by mitogen-activated protein kinase signaling (280). Some bacteria have also devised strategies to evade CD1d-restricted antigen presentation. Notably, the infection of monocytes by the human pathogen *M. tuberculosis* results in reduced CD1d mRNA expression, indicating the transcriptional control of *Cd1d* expression by a mycobacterial product (281).

While pathogens evade NKT cell activation by way of interference with intracellular CD1d trafficking and, thereby, antigen presentation, pathogens induce MAIT cell dysfunction to evade MAIT cell response. To that end, patients with *S. pneumoniae*–induced sepsis show significantly reduced but more active and dysfunctional MAIT cell responses compared to healthy donors or paired 90-day samples (139). The hyperactive MAIT cells stir up a pathological cytokine storm thought to be responsible for mortality (141). Furthermore, the hyperactive MAIT cell response poorly induces the differentiation of inflammatory monocytes to dendritic cells during pulmonary infection (139). Similarly, studies of *C. difficile* pathology indicate that these bacteria potently activate MAIT cells in a combined TCR- and cytokine-dependent manner inducing a pathological cytokine storm. The resultant runaway inflammation perhaps enables *C. difficile* to overcome cellular barriers to potentiate *C. difficile*–induced antibiotic-associated colitis (138). In a similar vein, gastric *H. pylori* infections elicit a hyperactive MAIT cell response, promoting an increased recruitment of inflammatory immune cells to the gastric mucosa exacerbating *H. pylori* gastritis (145). Thus, while some pathogens evade NKT cell recognition, the effects on MAIT cells focus on inducing MAIT cell hyperactivation and dysfunction as a means of potentiating bacterial pathogenicity.

## *Sic parvis magna*—greatness from small things come

Some 50 years ago, Ivan Riott and John Playfair and their respective groups, independently and a year or so apart, described a small subset of lymphocytes that were neither B nor T cells yet killed tumor cells without prior priming. While no small discovery in and of itself, it was a small beginning considering the numerous unconventional lymphocytes that were discovered in the ensuing decades. Unbeknownst, the discovery of NK cells had silently annunciated the existence of a grander system of cells whose constituents played critical roles in immunity to infectious diseases and cancer, as well as in precipitating autoimmune disorders and allergic reactions. Multitudinous, they are yet cluster together by several common phenotypic and functional features. Their purpose is to process and integrate signals received from the innate immune response to convey that umwelt to downstream innate and adaptive effector responses. In this manner, they appear to function in between, at the edges of the innate and adaptive immune systems. Hence, innate/innate-like effector lymphocytes are called in-betweeners—or, alternatively, Latinate edge, and a ‘limbic immune system’ arises, perchance. In this proposal for a triumvirate immune system, we do not insinuate that the ‘limbic immune system’ is an evolutionary transition between the innate and adaptive systems because the independently acting modules that make up this system arise at different times in evolution, repurposing loosely common genome regulatory circuits to accomplish a common task. The ‘limbic immune system’ functions to integrate information relayed by the innate sensory immune system about the local tissue environment and to provide context to downstream effector innate and adaptive immune responses. The multiple modules add robustness and evolvability to this limbic system to keep abreast of the ever-changing environment and the quick-evolving microbes, especially of those members of an otherwise symbiont community that turn pathobiont without much notice.

## Author contributions

SJ, GO and JD wrote and edited the MS. All authors contributed to the article and approved the submitted version.
